# Supplementary material for: Yiai Fuzheng decoction inhibits triple-negative breast cancer by remodeling the immune microenvironment
Source: Front Immunol. 2025 Sep 30;16:1615631. doi: 10.3389/fimmu.2025.1615631 (PMC12518410; doi:10.3389/fimmu.2025.1615631)
Supplement: Supplementary file 2 [file DataSheet1.zip › Supplementary File 2/Treatment/C_vs_H/07_Pathway_Analysis/Pathway_Analysis/Pathway_Bubbleplot.pdf]

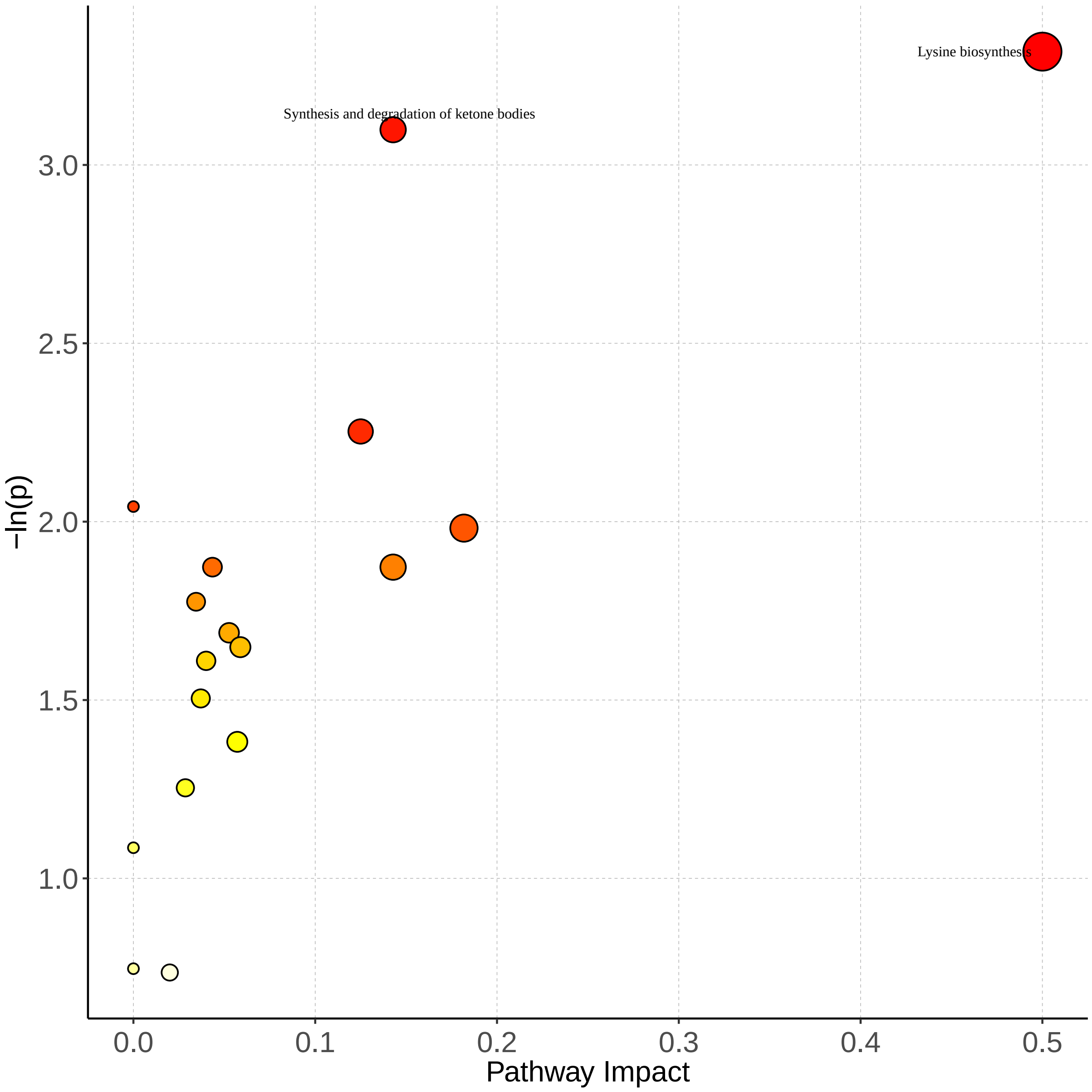
Lysine biosynthesis
Synthesis and degradation of ketone bodies
3.0
2.5
2.0
1.5
1.0
−ln(p)
0.0
0.1
0.2
0.3
0.4
0.5
Pathway Impact
